# Ultrasonographic monitoring of feline epaxial muscle height as part of an annual wellness examination to assess for the development of sarcopenia

**DOI:** 10.1177/1098612X221140081

**Published:** 2023-01-27

**Authors:** Aaron Lutchman, Natasha Shanker, Eithne Comerford, Alexander J German, Y Becca Leung, Thomas Maddox, Nathalie Dowgray

**Affiliations:** 1 School of Veterinary Science, University of Liverpool, Liverpool, UK; 2 Institute of Life Course and Medical Sciences, University of Liverpool, Liverpool, UK; 3 The MRC-Versus Arthritis Centre for Integrated research into Musculoskeletal Ageing (CIMA), Liverpool, UK; 4 Royal Canin Research Centre, Aimargues, France

**Keywords:** Mature, sarcopenia, muscle condition, muscle measurement

## Abstract

**Objectives:**

The aim of this study was to determine if epaxial muscle height (EMH) could be reliably incorporated into annual routine wellness screenings, and also determine its relationship to age, body condition score (BCS), subjective muscle assessment (SMA), breed and sex in mature cats.

**Methods:**

EMH was determined independently by three observers from ultrasonographic examinations – collected by an additional trained individual – of cats enrolled at the Feline Healthy Ageing Clinic, University of Liverpool, UK. Age, body weight, BCS and SMA data were also collected.

**Results:**

A total of 92 cats were included, 35 of which had repeat ultrasonographic examinations 12 months apart. Enrolled cats were a median age of 8 years and 9 months at the time of the first measurement. Variation in the quality of ultrasonographic images collected did not affect muscle depth measurements (*P* = 0.974). Further, there was good intra- and inter-observer repeatability for all observations (intraclass correlation range 0.97−0.99). There was a moderate positive association between EMH and body weight (*r* = 0.49, *P* <0.001) but no association with age (*r* = −0.05, *P* = 0.680). There were also positive associations in EMH among cats with different BCSs (*P* = 0.001) and SMAs (thoracic spine, *P* = 0.021; lumbar spine, *P* = 0.014), but breed (*P* = 0.429) and sex (*P* = 0.187) had no effect. Finally, there was no change in EMH measurements in the paired samples (*P* = 0.145) or correlation between percentage weight and EMH change over 12 months.

**Conclusions and relevance:**

The accuracy of EMH measurement using ultrasonographic imaging is good, irrespective of observer experience and provided that the ultrasonographer has some training. This suggests that ultrasonographic measurement of EMH could have a major practical impact as a non-invasive determination of muscle mass in pet cat populations. Further research is required to assess longitudinal changes in muscle mass over time in senior pet cats.

## Introduction

Sarcopenia is a term that was first proposed by Rosenberg in 1988 from the Greek *sarx*, meaning flesh, and *penia*, meaning loss.^1^ It is defined as the progressive, age-related loss of muscle condition and strength which, in turn, is associated with adverse health-related outcomes in older people.^2^ In the 2018 operational definition of sarcopenia in humans, ‘likely sarcopenia’ is identified by low muscle strength, ‘sarcopenia’ is confirmed by the presence of low muscle quantity or quality and ‘severe sarcopenia’ is used for cases meeting the above two criteria in addition to low physical performance.^3^ This is a change from an earlier consensus that put loss of muscle mass alone as the first sign of sarcopenia.^4^ The predominant feature of primary sarcopenia is that it occurs in the absence of any other definable cause.^3^ Secondary sarcopenia can occur owing to systemic disease, physical inactivity (due to a sedentary lifestyle or disease-related immobility) and inadequate energy or protein intake.^3^ Reversal of sarcopenia in ageing humans can be difficult; therefore, early intervention with appropriate nutritional support and exercise is important to slow its progression.^5^

There is currently a paucity of data regarding the identification, quantification and treatment of sarcopenia in cats. Muscle wasting has been reported in 22% of cats aged 7–10 years, 54% of cats aged 11–14 years and 77% of cats >15 years of age.^6^ In ageing cats, weight loss is associated with shorter survival times when chronic disease (eg, cancer, kidney disease and hyperthyroidism) is present; however, clinical assessment of sarcopenia in cats is not currently standardised.^7,8^ Weight measurement alone is a poor measure of sarcopenia^9^ but can be used clinically in conjunction with body condition scoring and muscle condition scoring. Both of these measurements have been validated against dual-energy X-ray absorption (DEXA) in cats,^10,11^ and can be used to identify possible changes in body composition and muscle wastage, respectively.^12^ However, an additional non-invasive method allowing for measurements of muscle mass would be helpful for clinicians, because both body condition score (BCS) and muscle condition score (MCS) are subjective estimations.^12^

Ultrasonography is an imaging modality that is gaining increasing recognition as a useful method for the assessment of epaxial musculature in companion animals, with attempts to standardise measurements.^13–15^ Ultrasonographic measurements of the epaxial muscle height (EMH) at the level of the 13th thoracic vertebrae were positively correlated with body weight in clinically normal cats (aged 1−6 years, BCS 4–6/9),^14^ while MCS was positively correlated with EMH in cats of various ages (mean 10.3 ± 3.9 years).^15^ However, to date, there have been no studies investigating either the use of this methodology as part of a routine wellness appointment or associations between EMH and different variables in mature pet cats (aged 7–10 years).^16^

Therefore, the first aim of this study was to determine if ultrasonographic EMH measurements are repeatable among observers and could readily be incorporated into a routine wellness screening consultation for mature cats. The second aim was to determine if EMH measurements were associated with variables such as age, body weight, BCS, subjective muscle assessment (SMA), breed and sex in mature cats, and also whether EMH measurements changed over time.

## Materials and methods

### Selection criteria

Cats attending the Royal Canin Feline Healthy Ageing Clinic (FHAC) at the University of Liverpool, between 2017 and 2020 were included at their enrolment or subsequent annual examinations. At enrolment to the FHAC, all owners were provided with a detailed information sheet explaining the purpose of the study before giving written consent for their cat to be enrolled. Eligibility criteria included a temperament that was amenable to having a conscious ultrasonographic examination; additional criteria for enrolment to the FHAC included age (7–9 years).^17^ Cats were excluded where recorded cine-loop ultrasonographic images were not of a sufficient quality to allow measurement of the epaxial muscles using predefined landmarks. Ethical approval for this study was granted by the University of Liverpool Veterinary Research Ethics Committee (VREC491abcd) and the Royal Canin Ethical Committee.

### Clinical assessment

Baseline data, including age, sex and breed, were recorded at enrolment. Body weight was measured using the same set of electronic scales (V20; Burtons), which were calibrated on a weekly basis using 2.5 kg fitness weight plates. To assess BCS, a validated nine-unit system was used,^18^ while SMA was based on a previously validated system whereby a score of 3 indicated no muscle wastage, a score of 2 indicated mild muscle wastage, a score of 1 indicated moderate muscle wastage and a score of 0 indicated marked muscle wastage.^10^ However, to distinguish scores from those of BCS, the terminology used for the categories was that recommended by the World Small Animal Veterinary Association: 3 to 0 were replaced with A to D.^19^ Although SMA was assessed at 10 different points of the body, for the purposes of this study, only the scores for the epaxial muscles (thoracic spine and lumbar spine) were used rather than a global score, as this is where muscle loss is first typically noticed in cats.^19^ Body weight, BCS and SMA were collected by the investigator (ND) at the time of each ultrasonographic assessment.

### Ultrasonographic assessment

All ultrasonographic examinations were performed by ND, a veterinarian and PhD student at the FHAC, who had been trained by an EBVS European Specialist in Veterinary Diagnostic Imaging (TM). Training consisted of a 40 min session optimising the settings on the ultrasound machine and standardising the protocol for the ultrasonographic probe placement. Cats were either held in a standing position or positioned in sternal recumbency. The hair was parted over either the left or right thoracolumbar junction, with the side of the cat that was imaged determined solely by the cats’ comfort and posture. Before measurements were taken, ultrasound gel (blue ultrasound gel; Henleys Medical Supplies) was used to saturate the fur, and a 75L38 5–10 MHz 38 mm footprint linear probe (Mindray DP-50; IMV) was then used to collect measurements. Landmarks for ultrasonographic probe placement were determined by palpating the junction of the 13th rib and vertebrae. Multiple ultrasonographic cine-loops were recorded and saved as digital imaging and communications in medicine (DICOM) files for analysis.

**Figure 1:**
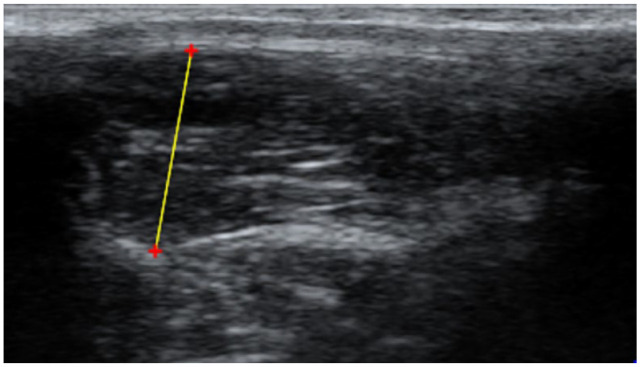
Measurement of epaxial muscle height in mature cats using ultrasonography. A cursor was placed ventromedial to the muscle, at the interface between the 13th rib or vertebrae and the muscle. A second cursor was placed dorsolateral, at the interface between the subcutaneous fat and the muscle. Red ‘+’ are cursors and the yellow line represents a distance measurement (mm)

The ultrasonographic cine-loops were first assessed for visibility of landmarks for EMH measurement by observer 1 (AL; a veterinarian) and observer 2 (NS; a final-year veterinary student). Landmarks assessed for visibility were the junction of the 13th rib and vertebrae, and the junction of the muscle and subcutaneous fat. If the required landmarks were not visible, the cine-loops were discarded. For cats with multiple cine-loops, the best-quality cine-loop (ie, the one that showed the required landmarks most clearly) was selected, and three measurements were made using the measurement callipers of a freely available DICOM viewer (Horos Project version 4.0.0). For each measurement, one cursor was positioned at the junction of the 13th rib–vertebral junction and the ventromedial aspect of the epaxial muscle; the second cursor was positioned at the junction between muscle and subcutaneous fat at the dorsolateral aspect of the muscle (Figure 1), as has been previously described.^14,15^ For each observer, the mean muscle height was calculated from the three measurements taken from each cat, as previously described.^14,15^

### Method validation

Observer 2 independently graded all cine-loops for quality using a 3-point grade scale, following consultation with ND and TM. High-quality images, where anatomical landmarks were fully visible, were graded 1; reasonable quality images, where some areas of the cine-loop were unclear but landmarks were still visible, were graded 2; and poor-quality images, where the landmarks were not fully visible but measurements could still be made, were graded 3. To determine intra- and inter-observer association and agreement in scoring, observers 1 (veterinarian) and 2 (veterinary student) measured EMH from the ultrasonographic cine-loops, following a short period of training by TM. Additionally, a third observer (observer 3; EBVS European specialist in veterinary diagnostic imaging [TM]) also assessed 30 cine-loops (validation image set), selected from all the cases using a random number generator (Microsoft Excel version 16.47).

### Data handling and statistical analysis

Statistical analysis was performed using an online open-access statistical language and environment (R, version 4.11; www.R-project.org) and the following packages: ‘ggplot 2’, ‘ggpur’, ‘tidyverse’ and ‘psych’. Data sets were assessed for normality using the Shapiro–Wilk test and visual assessment of histograms. Coefficient of variation (CV) was used as a measure of relative variability in the individual observer measurements. Parametric tests were used for normally distributed data, with results presented as mean ± SD. Non-parametric tests were used for data that were not normally distributed, with results presented as median (range). The level of statistical significance was set at *P ⩽*0.05, and all reported *P* values are two-sided.

Bland–Altman plots were created to assess the agreement of measurements between observers. Intra- and interclass correlation coefficients were calculated, a two-way mixed-effects model based on single rating for the intra-observer calculation and a two-way random-effect model based on single ratings and absolute agreement for inter-observer calculations were used.

Using the primary image set (first images collected from each cat, regardless of quality and not including any repeat measures), associations between mean EMH measurements (mean EMH was calculated from all six measurements from both observers 1 and 2) and continuous baseline variables (age and body weight) were examined using scatter plots and Pearson’s correlation coefficient. The effect of categorical variables (BCS, SMA and sex) on mean EMH and of CV in different image-quality categories were assessed with box plots in conjunction with either Kruskal–Wallis tests (BCS and CV in different quality grades), Mann–Whitney tests (SMA [scores A vs B]) or unpaired *t*-tests (sex [male vs female] and breed [pedigree vs domestic]). Finally, where repeat measurements 12 months apart were available, change in mean EMH, body weight and BCS were assessed, using either paired *t-*tests or Wilcoxon signed rank tests when the data were parametrically or non-parametrically distributed, respectively. The relationship between changes in body weight and EMH was examined using scatter plots and Kendall’s tau (Kendall rank correlation coefficients).

## Results

### Study animals and ultrasonographic cine-loop image sets

Ultrasonographic cine-loops were initially available from 94 cats, but cine-loops from two cats were excluded because the required anatomical landmarks were not visible; therefore, sets of cine-loops available from 92 cats were included in this primary image set. Repeat measurements, 12 months apart, were also available for 35 cats (secondary image set). There were 78 domestic cats (long- and shorthair), 14 pedigree cats (three Persians; two Ragdolls; two Siamese) and individual cats from seven other breeds (American Exotic, Bengal, Egyptian Mau, Maine Coon, Oriental, Russian Blue and Sphynx). There were 49 male cats (48 neutered) and 43 female cats (all neutered), while the median age at first measurement was 105 months (8 years and 9 months; range 80–143 months), median body weight was 4.8 kg (range 3.1–9.2) and median BCS was 6/9 (range 3–9). Epaxial SMA was graded B in 6/92 cats over the thoracic spine and 7/92 cats over the lumbar spine at first assessment, and 1/35 cat at the second assessment; all other cats received a SMA of A at both locations and time points. Mean EMH was 13.6 ± 2.60 mm at first assessment and 13.9 ± 2.49 mm at the second (Table 1).

**Table 1:** Baseline data and mean epaxial muscle height measurements in cats at different time points

Descriptor	Image set	
	Primary (n = 92)	Secondary (n = 35)
Age (months)	105 (80–143)	113 (85–145)
Weight (kg)	4.8 (3.1–9.2)	4.8 (3.2–7.2)
BCS	6 (3–9)	6 (3–9)
SMA-LS	A (A–B)	A (A–B)
SMA-TS	A (A–B)	A (A–B)
EMH (mm)	13.6 ± 2.60	13.9 ± 2.49

Data are median and range for all variables except epaxial height measurements (EMH), which are reported as mean ± SD

BCS = body condition score; SMA-LS = subjective muscle assessment measured over the lumbar spine; SMA-TS = subjective muscle assessment measured over thoracic spine

### Agreement among observers

**Figure 2:**
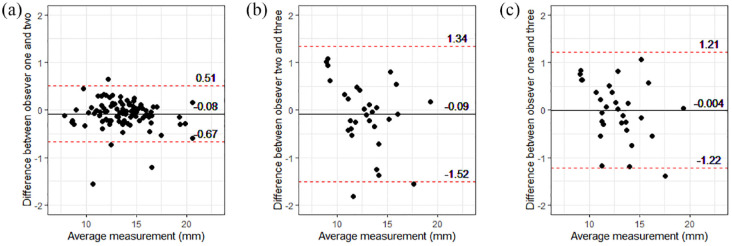
Bland–Altman plots showed inter-observer agreement in epaxial muscle height (EMH) measurements was excellent, with no evidence of systematic or proportional bias. (a) Inter-observer agreement between observer 1 (veterinarian) and observer 2 (veterinary student) on measurements from 92 mature cats. (b) Inter-observer agreement between observer 2 (veterinary student) and observer 3 (EBVS European specialist in veterinary diagnostic imaging) on 30 measurements from 30 mature cats. (c) Inter-observer agreement between observer 1 (veterinarian) and observer 3 (EBVS European specialist in veterinary diagnostic imaging) on 30 measurements from 30 mature cats. Individual measurements are shown by black filled circles, the solid black line represents the mean difference between observers (bias) and the red dotted lines show the 95% limits of agreement

When assessing intra-observer variability (Table 2), relative variability was low in observers 1 (veterinarian: median CV 1.9 [range 0.2–8.3]) and 2 (student: median CV 1.7 [range 0.4–6.3]). Additionally, intra-observer agreement in EMH measurements was excellent for both observers 1 and 2 (Table 2). Visual inspection of Bland–Altman plots revealed excellent inter-observer agreement with no obvious systematic or proportional bias (Figure 2). Between observers 1 and 2 (veterinarian vs student), the mean bias was −0.08 mm (95% limits of agreement [LoA] 0.51 to −0.67; Figure 2a); between observers 2 and 3 (student and imaging specialist), the mean bias was −0.09 mm (95% LoA 1.34 to −1.52; Figure 2b), while between observers 1 and 3 (veterinarian vs imaging specialist), the mean bias was −0.004 mm (95% LoA 1.21 to −1.22; Figure 2c). The median absolute magnitude of difference between observers 1 and 2 was 0.140 mm (range 0.003−1.740); it was 0.402 mm (range 0.023−1.393) between observers 2 and 3 and 0.402 mm (range 0.020−1.820) between observers 1 and 3. Furthermore, there was good inter-observer agreement between observers 1 (veterinarian) and 2 (student), and good inter-observer repeatability among all three observers (intraclass correlation coefficient 0.97 [95% confidence interval, 0.95−0.98]; *P* <0.001; Table 2).

**Table 2:** Agreement between observers for epaxial muscle ultrasound height measurements

	ICC	95% CI	*P* value	Median CV	Range
Intra-observer*					
Observer 1	0.98	0.98–0.99	<0.001	1.9	0.2–8.3
Observer 2	0.98	0.98–0.99	<0.001	1.7	0.4–6.3
Inter-observer					
Observer 1 vs 2	0.99	0.99–1.00	<0.001	2.5	0.7–9.3
Observer 1 vs 2 vs 3	0.97	0.95–0.98	<0.001	1.9	0.5–7.9

* Observer 1, veterinarian; observer 2, veterinary student; observer 3, veterinary diagnostic imaging specialist

ICC = intraclass correlation; CI = confidence interval; CV = coefficient of variation

### Ultrasonography cine-loop quality

**Figure 3:**
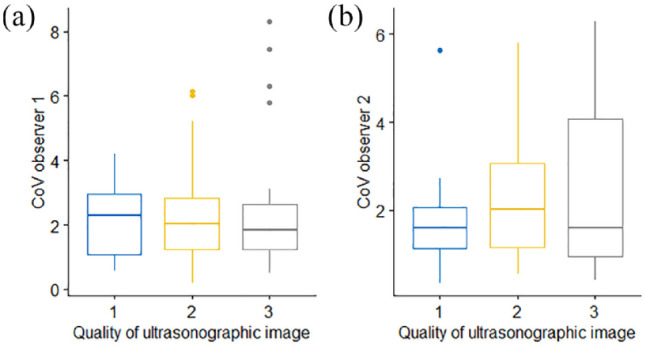
Box and whisker plots depicting the coefficient of variation (CV) of epaxial muscle height measurements from 92 cats, taken by (a) observer 1 and (b) observer 2, and subdivided by image quality, graded from 1 (best) to 3 (worst) quality. The thick horizontal lines depict the median, the boxes depict the interquartile range and the whiskers represent the 95% range, with outliers being depicted as filled circles. Variability in muscle height measurements did not differ between cine-loops of different quality. (a) *P* = 0.961; (b) *P* = 0.546

Mean EMH measurements did not differ among different quality grades of ultrasonographic images in the primary image set (*P* = 0.974). Variability in muscle height measurements did not differ between cine-loops of different quality when assessed by both observer 1 (*P* = 0.961; Figure 3a) or observer 2 (*P* = 0.546; Figure 3b).

### Associations between EMH and other variables

**Figure 4:**
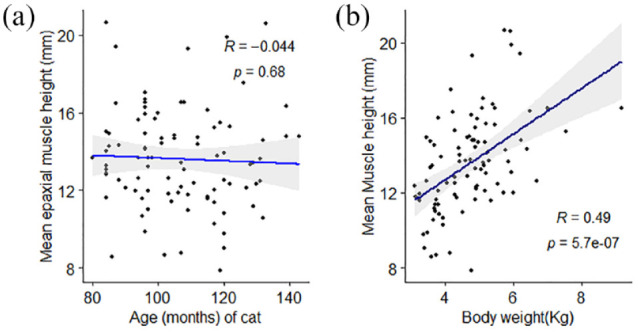
Associations between mean feline epaxial muscle height (EMH) measurements and (a) age or (b) body weight in 92 mature cats. Individual measurements are shown by black filled circles, the black line represents the line of best fit and the grey shaded region represents the 95% confidence interval. There was a moderate positive correlation between EMH measurements and body weight but not age

**Figure 5:**
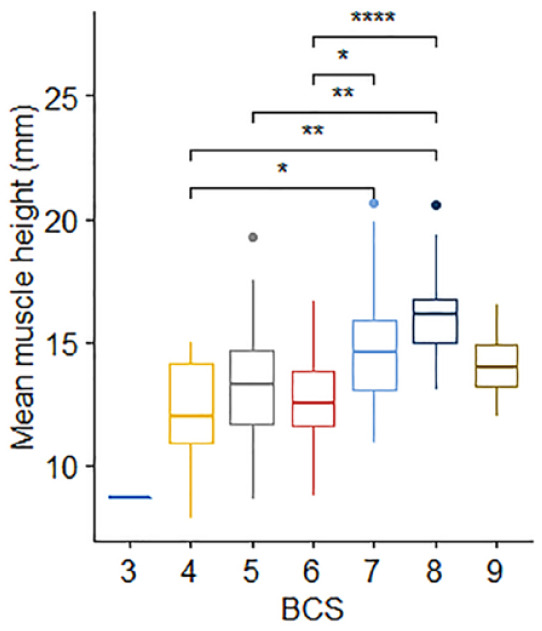
Epaxial muscle height (EMH) measurements (mm) in 92 mature cats, stratified by body condition score (BCS). The thick horizontal lines depict the median, the boxes depict the interquartile range, the whiskers represent the 95% confidence interval and outliers are depicted as filled circles. Cats with a BCS of 8/9 had an EMH significantly greater than the EMH of cats with a BCS of 4/9 (*P* = 0.001), 5/9 (*P* = 0.001) or 6/9 (*P* <0.001). Cats with a BCS of 7/9 had an EMH significantly greater than the EMH of cats with a BCS of 4/9 (*P* = 0.048) and 6/9 (P = 0.012). *P = 0.01−0.05; **P = 0.001−0.01; ****P <0.001

**Figure 6:**
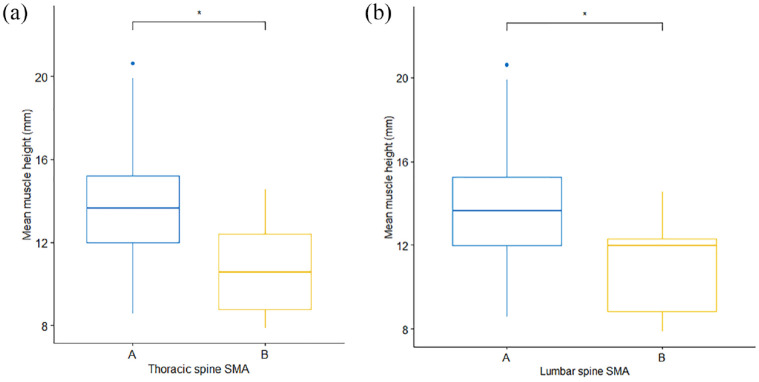
Epaxial muscle height (EMH) measurements (mm) in 92 mature cats, stratified by subjective muscle assessment (SMA) and assessed over both the (a) thoracic and (b) lumbar spine. The thick horizontal lines depict the median, the boxes depict the interquartile range, the whiskers represent the 95% confidence interval and outliers are depicted as filled circles. Cats with normal muscle mass were assigned an SMA score if A (blue), while those with mild muscle wasting were assigned a score of B (yellow). At both sites, mean muscle height measurements were less in cats with SMA B than in those with SMA A (*P* = 0.021 and *P* = 0.014, respectively). *P = 0.01−0.05

In the primary image set, there was no significant difference in mean EMH between cats of different sex (*P* = 0.187) or domestic vs pedigree breed (*P* = 0.429). No correlation was observed between EMH and age (*r* = −0.05, *P* = 0.68; Figure 4a), but there was a moderate positive correlation between EMH and body weight (*r* = 0.49, *P* <0.001; Figure 4b). Mean EMH was significantly greater in cats with a BCS of either 7 or 8 than in cats with a BCS of 4 or 6; furthermore, cats with a BCS of 8 had a significantly greater mean EMH than cats with a BCS of 5 (*P* = 0.001; Figure 5). Mean EMH was significantly greater in cats with an SMA of A compared with SMA B for both the thoracic spine (*P* = 0.021) and lumbar spine (*P =* 0.014) regions (Figure 6).

### Variation in cats over a period of 12 months

**Figure 7:**
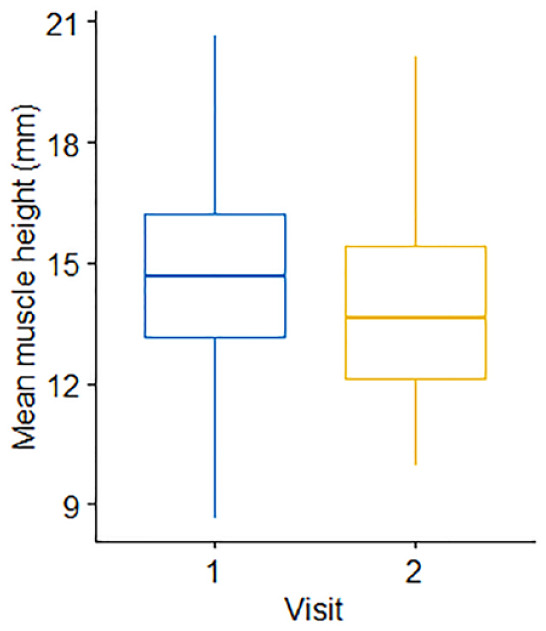
Comparison of mean epaxial muscle height (mm) measurements in 35 cats, 12 months apart. The thick horizontal lines depict the median, the boxes depict the interquartile range and the whiskers represent the 95% confidence interval. Visit 1 denotes the initial measurement; visit 2 denotes the repeat measurement taken 12 months later. There was no change in muscle height over 12 months (*P* = 0.15)

**Figure 8:**
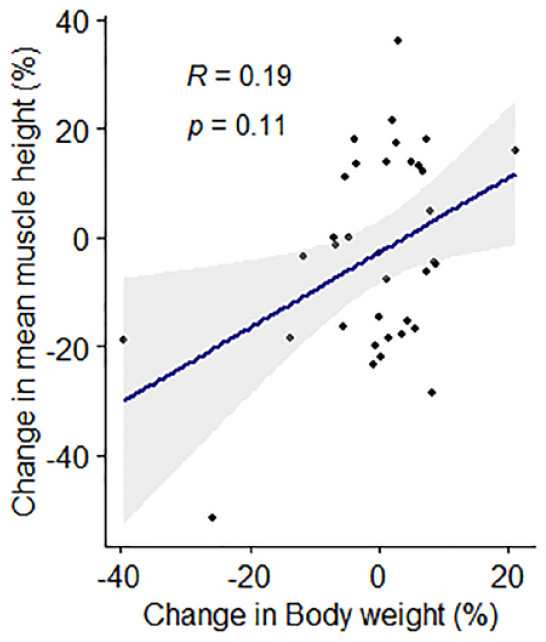
Associations between percentage change in feline epaxial muscle height (EMH) measurements and percentage change in body weight over 12 months in 35 mature cats. Individual measurements are shown by black filled circles, the dark blue line represents the line of best fit and the grey shaded region represents the 95% confidence interval. There was no correlation between percentage change in EMH measurements and percentage change in body weight

In the cats (n = 35) with repeat assessments at 12 months apart, no significant differences in either body weight (*P* = 0.474) or BCS (*P* = 0.516) over time were recorded. No significant difference was observed in median EMH between the first (14.7 ± 2.82 mm) and second (13.9 ± 2.48 mm) measurements (*P* = 0.145; Figure 7); mean change in EMH was −0.8 ± 3.02 mm. Median change in individual cat body weight for the 35 cats was 0.07 kg (range −3.63 to 0.91 kg); no correlation was observed between percentage change in body weight and EMH (*r* = 0.19, *P* = 0.110; Figure 8). SMA was unchanged in all but two of the 35 cats (6%), where SMA improved from B to A at both sites for both cats. In one of these cats, mean EMH increased from 12.51 mm to 14.49 mm, while weight (from 4.29 to 5.2 kg) and BCS (from 6 to 7) also increased; in the second cat, mean EMH decreased from 14.55 mm to 11.83 mm, while weight (from 3.70 to 3.19 kg) and BCS (from 5 to 4) also decreased.

## Discussion

Findings from this study support those of other studies, where a correlation between EMH and body weight, and also between EMH and SMA, has been reported.^14,15^ Together, these studies support the potential for ultrasonographic assessment of muscle loss, providing an additional valuable tool for assessing the health of ageing cats.

This is the first study to look at repeat measurements of EMH in cats. There was no significant decline in EMH over a period of 12 months and further longitudinal assessment of EMH in cats is required.

There were also differences in EMH in cats with different BCSs, whereby cats with greater body fat mass (BCS 7 or 8) had a significantly greater EMH than cats with less body fat mass (eg, BCS 4−6). This is, perhaps, not unexpected because lean tissue mass is greater in cats with obesity.^11^ However, this increase in EMH might also be due to compensatory fat deposition within and around myocytes, as seen in elderly people (>70 years), especially women, with an increased body fat mass.^20,21^ This condition is called ‘sarcopenic obesity’, and is associated with an increased risk of compromised mobility.^4,21^ Similar findings have been shown to occur in cats with obesity,^22^ and may compromise identification of sarcopenia using either SMA or ultrasonography. Advanced diagnostic imaging modalities, such as CT or MRI, would be required to assess such intramuscular fat deposition.^21^

Both intra- and inter-observer ICC were good, suggesting good agreement between observers with different levels of training, from a veterinary student to a diagnostic imaging specialist. This finding is also supported by Bland−Altman plots, whereby the 95% LoAs were 0.51 to −0.67, 1.34 to −1.52 and 1.21 to −1.22, respectively. Given these findings and the fact that image quality had limited impact on EMH results, EMH measurements could reliably be used to monitor cats in primary care practice, provided that veterinary professionals receive a modest amount of appropriate training. However, a limitation of this study is that all EMH measurements were performed on one set of images collected by a single examiner. Variability may be greater within and between observers if they had to collect images themselves or they were measuring images collected by several different examiners.

There was no significant correlation in mean EMH with age, which may have been due to the small age range of the cats enrolled. No cats with severe muscle loss were included in this study. Muscle strength or performance was not assessed, so the physiological significance of sarcopenia in cats with mild muscle wastage could not be fully evaluated. As sarcopenia is an age-related condition, further study with a larger cohort of older animals and greater range of ages would therefore be useful.

In addition to the narrow age range, other limitations associated with the demographics of the study population include the small number of pedigree cats and also those with moderate or severe muscle wastage. Documenting comorbidities would be critical to interpreting sarcopenic effects clinically.^23^

Additional limitations include the operator skill during ultrasonographic image collection, as manual manipulation risks excessive muscle compression (artefactually reducing muscle height) during the examination and may also compromise the identification of anatomical landmarks for accurate measurements.^15^ However, this study demonstrated that repeatable results could still be obtained from poorer-quality images. Nevertheless, several images were removed from the data set as landmarks could not be identified, creating bias in the data set. The training required for EMH measurement is straightforward, including the appropriate application and direction of the ultrasound probe, as well as identification of the predefined landmarks. Posture and inconsistency when measuring the left side or right side of the spine would preferably be standardised, although another study demonstrated no difference in measurements between standing or crouching and left or right epaxial muscle measurements.^14^ Finally, normalisation of EMH to body size was not performed. Normalisation has been previously described in cats using the forelimb circumference measurement,^14,15^ and will be useful to include in future work where comparison between cats is required.

## Conclusions

Measurements of EMH were associated with body weight, BCS and SMA but not with age, breed or sex in this cohort of cats. There was no relationship between changes in weight and EMH in individual cats over 12 months, but further longitudinal research on the changes to epaxial muscle depth measurements as individual cats age is warranted. This study suggests that ultrasonographic measurement of EMH could have a major practical impact as a non-invasive determination of muscle mass in pet cat populations.
